# Characteristics and outcomes of in vitro fertilization in different phenotypes of polycystic ovary syndrome

**DOI:** 10.4274/tjod.90094

**Published:** 2016-03-10

**Authors:** Selçuk Selçuk, Enis Özkaya, Ahmet Eser, Melda Kuyucu, Hüseyin Tayfun Kutlu, Belgin Devranoğlu, Kenan Sofuoğlu, Vedat Erkan Dayıcıoğlu

**Affiliations:** 1 Zeynep Kamil Women and Children’s Diseases Education and Research Hospital, Clinic of Obstetrics and Gynecology, İstanbul, Turkey; 2 Medistate Hospital, Clinic of Obstetrics and Gynecology, İstanbul, Turkey

**Keywords:** Polycystic ovary syndrome, IVF, phenotypes

## Abstract

**Objective:**

The aim of this study was to investigate whether polycystic ovary syndrome (PCOS) phenotype without polycystic ovaries (PCO) differs in terms of in vitro fertilization (IVF) outcomes compared with classic phenotypes.

**Materials and Methods:**

This retrospective controlled study included 262 patients who underwent IVF treatment with an indication of unexplained or tubal factor infertility (control group), ovulatory patients with PCO morphology (group 1), PCOS phenotype with oligoanovulation and hyperandrogenemia (group 2), PCOS phenotype with PCO morphology and oligoanovulation (group 3). Outcomes and baseline characteristics of IVF-embryo transfer treatments were compared among all groups.

**Results:**

PCOS phenotype without PCO morphology had similar IVF stimulation characteristics compared with classic phenotypes; however, a higher total gonadotropin dose was needed to achieve similar results compared with patients with PCO morphology with or without PCOS. Basal follicle-stimulating hormone level (beta coefficient=0.207, p=0.003), group (beta coefficient=-0.305, p<0.001) and age (beta coefficient=0.311, p<0.001) were significantly associated with the total gonadotropin dose. The number of good quality embryo on transfer day was significantly lower in patients with isolated PCO morphology and PCO morphology with oligoanovulation than in those with PCOS phenotype without PCO morphology.

**Conclusion:**

PCO morphology provides easier stimulation, whereas hyperandrogenemia provides better results as good quality embryos. However, the end point is similar in terms of biochemical, clinical, and ongoing pregnancy rates.

## INTRODUCTION

Polycystic ovary syndrome (PCOS) is a complex disorder and may present in different phenotypes. Previous data included only the classic phenotype characterized by chronic anovulation and hyperandrogenism^(1,2,3)^. However, accumulated data led to the Rotterdam criteria, which allows for different PCOS phenotypes^(4)^. According to the defined criteria, four different phenotypes can be introduced: I. Hyperandrogenism, chronic anovulation, and polycystic ovaries (PCO); II. Hyperandrogenism and chronic anovulation but normal ovaries; III. Hyperandrogenism and polycystic ovaries but ovulatory cycles; and IV. Chronic anovulation and polycystic ovaries but no clinical or biochemical hyperandrogenism. A meta-analysis concluded that unknown intra-or extraovarian abnormalities may interfere with granulosa cell-oocyte interaction, oocyte maturation, and potential embryonic development and result in unsuccessful artificial reproduction techniques in PCOS^(5)^. However, it is not known whether different phenotypes of PCOS have similar results. It is well known that PCOS is associated with elevated adrenal androgens such as dehydroepiandrosterone sulphate (DHEAS) in 20-50% of cases and meta-analyses about the effect of DHEAS supplementation in assisted reproduction revealed improvement of oocyte production and pregnancy rates^(6,7,8,9)^. However, according to the aforementioned data, we know that all PCOS phenotypes do not have hyperandrogenemia or even PCO morphology, so is it possible to generalize all in vitro fertilization (IVF) outcomes in PCOS by assessing a mixture of women with different PCOS phenotypes?

In this study, we aimed to assess IVF characteristics among different PCOS phenotypes to show whether PCO morphology or hyperandrogenemia would interfere with the results.

## MATERIALS AND METHODS

### Study population

Between 2009 and 2014, among the women referred to the infertility unit of the Department of Obstetrics and Gynecology, Zeynep Kamil Women and Children’s Health Training and Research Hospital, 262 patients who underwent IVF treatment with an indication of unexplained or tubal factor infertility (control group, n=84), ovulatory patients with isolated PCO morphology (group 1, n=85), PCOS phenotype with oligoanovulation and hyperandrogenemia (group 2, n=38), PCOS phenotype with PCO morphology and oligoanovulation (group 3, n=55) were enrolled in the study (Table 1). Sample size was calculated according to the study by Kim et al.^(10)^ with 95% confidence interval (CI) and 80% power. The exclusion criteria were age ≥40 years; body mass index (BMI) >35 kg/m^2^; basal follicle-stimulating hormone (FSH) level >12 mIU/mL; presence of endometriosis; three or more previous unsuccessful IVF treatment; and systemic illness or endocrine disorders such as hyperprolactinemia, hypothyroidism, Cushing’s syndrome and non-classic congenital adrenal hyperplasia. The groups were compared in terms of basal clinical characteristics and IVF outcomes. PCO morphology was determined based on the data from transvaginal ultrasound showing the presence of 12 or more peripherally-oriented cystic structures in one ultrasonographic plane, each of which measured 2 to 10 mm in diameter^(11)^. Oligo-anovulation was described as progesterone <3 ng/mL (<9.54 nmol/L) on days 18-21 and/or a menstrual cycle of longer than 35 days. Patients with an elevated serum testosterone >60 ng/dL (>2.08 nmol/L) and/or serum Δ4A levels >3.8 ng/mL were considered to have biochemical hyperandrogenemia and subjects with a Ferriman Gallwey score >8 were accepted as having clinical hyperandrogenemia^(12)^. The study protocol was approved by the Local Ethics Committee of Zeynep Kamil Research and Teaching Hospital.

### Treatment protocol

On cycle day 3, ovarian stimulation was started by daily injection of recombinant FSH (r-FSH) (Gonal-F, Merck Serono, İstanbul, Turkey) with a starting dose specific for cases according to their age, BMI, ovarian reserve, and antral follicle count (AFC). According to the antagonist protocol, gonadotropin-releasing hormone (GnRH) antagonist (Cetrotide; 0.25 mg; Merck Serono, İstanbul, Turkey) injection was started from the 6th day of stimulation. Monitorization of the cycles was based on the ultrasound examination to record the number and size of the follicles and the double-layer endometrial thickness. For each participant on cycle days 2-3, transvaginal ultrasound was performed to determine AFC and screen for ovarian cysts. A repeat examination was performed on day 6 of stimulation and subsequently every 1-3 days as clinically indicated until the criterion for subcutaneous administration of recombinant chorionic gonadotropin alpha 250 mg (Ovitrelle; Merck-Serono, İstanbul, Turkey) was reached; at least two follicles ≥17 mm in diameter. Ovum retrieval was performed 36 h later. In all cases, an intracytoplasmic sperm injection procedure was performed on the same day (day 0) and embryo transfer was performed on day 3, 4 or 5 based on the quality of embryos. From the day of ovum retrieval, the luteal phase was supported by progesterone intravaginally (Crinone 8% gel; Serono, İstanbul, Turkey) twice a day.

### Assessment of embryo quality

Embryo quality was described according to the blastomeres size and number, the degree of fragmentation, and the presence of multinucleated blastomeres. Embryos with 4 or 5 equal- sized blastomeres and less than 10% cytoplasmic fragmentation with no multinucleation were accepted as good quality on day 2. An embryo with 7 or 8 equal-sized blastomeres with less than 10% cytoplasmic fragmentation and no multinucleation was accepted as good quality on day 3. A good quality embryo on day 4 was determined with following characteristics: embryo cavitation with compacted properties without morphologic anomalies such as vacuolation, excessive fragmentation, large number of excluded cells, and self-cavitation of cells^(13,14)^. On day 5, blastocyst quality and expansion were described in accordance with the classification of Gardner and Schoolcraft^(15)^ and good quality embryo was accepted as ≥grade 3AA.

### In vitro fertilization treatment outcomes

Chemical pregnancy was defined as positive β-hCG test following embryo transfer. Clinical pregnancy was defined as presence of gestational sac with fetal cardiac activity under ultrasonography. An ongoing pregnancy was defined as a pregnancy ≥12 weeks of gestation confirmed with ultrasonographic examination. Data of pregnancy outcomes were obtained from the hospital database or the pregnant women via phone. The implantation rate was calculated by dividing the number of gestational sacs with fetal cardiac activity by the number of embryos transferred. The primary end-points of the study were the chemical pregnancy rate, the clinical pregnancy rate, and ongoing pregnancy rate. Secondary outcomes evaluated were the total gonadotropin dose used, estradiol (E2) level on the trigger day, peak estradiol level, number of dominant follicles, number of metaphase II (MII) oocytes, MII oocytes rate, and implantation rate.

### Statistical analysis

Statistical analysis was performed using SPSS version 11.5 software. All data were summarized using descriptive statistics, correlation analyses were used to show associations, multivariate regression was used to show adjusted associations, receiver operating characteric curve analysis was used to calculate predictive value, and the sensitivity and specificity. One-way ANOVA and Pearson’s Chi-square tests were performed where appropriate; p=0.05 was accepted as the degree of significance. Data were given as mean ± standard deviation or percentage.

## RESULTS

**Figure 1:**
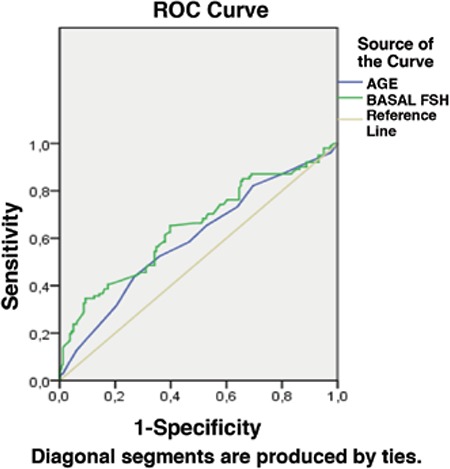
Receiver operating characteric curve of basal follicle stimulating hormone level and age to predict high gonadotropin dose

The baseline clinical characteristics of all groups are given in Table 2. There were no significant difference in terms of age, BMI, basal FSH, E2 levels, and duration of infertility among all groups. The characteristics of IVF cycles of patients are detailed in Table 2. The total gonadotropin dose was similar between group 2 and the control group, whereas it was significantly lower in groups 1 and 3 than in other groups (p_1_=0.002, p_2_<0.001, p_3_=0.006). Basal FSH level (beta coefficient=0.207, p=0.003), group (beta coefficient=-0.305, p<0.001) and age (beta coefficient=0.311, p<0.001) were significantly associated with the total gonadotropin dose. There was significant correlation between total gonadotropin dose and age (r=0.303, p<0.001), AFC (r=-0.553, p<0.001), basal FSH level (r=0.243, p<0.001), and group (r=-0.243, p<0.001). Age (AUC=0.595, =0.009) and basal FSH level (AUC=0.646, p<0.001) were found as significant predictors for the high gonadotropin dose >1800 IU, detrimented by the median level (Figure 1). The cut-off value for age was 29.5 years with 58% sensitivity and 54% specificity. The cut-off value for basal FSH was 6.2 with 65% sensitivity and 60% specificity. There were significantly higher numbers of oocytes retrieved in groups 1 and 3 when compared with patients in the control group; it was similar among groups 1, 2 and 3. There was no significant difference with respect to the ratio of MII oocytes and implantation rates among all groups. The number of good quality embryos on transfer day was significantly lower in patients in group 1 and 3 than in group 2. pregnancy rates are shown in Table 3. Biochemical, clinical and ongoing pregnancy rates were found similar among all groups.

## DISCUSSION

In the present study, we aimed to assess IVF characteristics among PCOS phenotypes with and without hyperandrogenemia or PCO morphology. Analyses of the data showed that IVF has similar success rates in patients with PCOS independent of presence or absence of hyperandrogenemia or PCO morphology. However, as expected, PCO morphology provided stimulation with lower doses and needed lower amounts of total gonadotropin dose. The number of good quality embryos was found to be significantly higher in the PCOS phenotype with oligoanovulation and hyperandrogenemia group. Despite the absence of PCO morphology, the numbers of dominant follicles were found comparable between this group and patients with PCO morphology. Good quality embryos and a comparable number of dominant follicles led us to conclude that hyperandrogenemia may have a favorable effect. However, this group needed a similar total gonadotropin dose when compared with the control group and higher total gonadotropin dose than the groups with PCO morphology. This shows that PCO morphology provides easier stimulation; hyperandrogenemia provides better results in terms of good quality embryos. Jamil et al.^(16)^ compared the clinical and hormonal parameters among four phenotypes of PCOS based on the Rotterdam criteria and with a control group. Women in the oligo-anovulation and PCO group and in the control group had significantly lower levels of luteinizing hormone/FSH ratio, total testosterone, and free androgen index, and higher levels of FSH and sex hormone-binding globulin when compared with women in the oligo-anovulation, PCO and hyperandrogenemia groups^(16)^. In the literature, it was stated that androgens were found to have a favorable effect on follicle maturation, especially during the early stages^(17)^. However, other studies showed a negative effect of androgens on folliculogenesis and embryonic development^(18)^. Androgens have been suggested to have a modulating effect on FSH activity in developing granulosa cells, and studies on PCOS have shown that androgens have a positive and negative effect on folliculogenesis^(17)^. Despite the changing effects of androgens and PCO morphology among groups, the end point is similar in terms of biochemical, clinical, and ongoing pregnancy rates. A recently published study on the effect of PCO morphology on oocyte quality in intracytoplasmic sperm injection cycles compared with a control group showed neither positive nor negative effects and the MII oocyte number was found to be higher in the group with PCO morphology, whereas the ratio of MII oocyte was similar, the number of top quality embryos was comparable between groups but the implantation and clinical pregnancy rates were found significantly higher in the PCO morphology group^(19)^. The authors tried to assess the effect of PCO morphology alone on oocyte quality so their results were not consistent with ours because of the ignored effect of hyperandrogenemia, which was shown to have a favorable effect in our study. In addition, patients with PCOS had similar IVF outcomes compared with other hyperresponders without PCOS in a study by O’Neill et al.^(20)^ Our study also produced similar end results but our data showed some critical cycle characteristics among different PCOS phenotypes. Ryan et al.^(21)^ showed a negative effect of prolonged stimulation in assisted reproductive technology cycles but the authors claimed that this effect was not observed in patients with PCOS. According to their article, PCOS had a different response to stimulation in a wide range, and to the best of knowledge, different phenotypes were not assessed separately. The effect of basal testosterone levels in IVF cycles of patients without PCOS was evaluated in a study by Sun et al.^(22)^ consistent with our results in PCOS patients, the authors concluded that although basal testosterone did not predict pregnancy outcomes, it was associated with the large follicles on human koryonik gonadotropin day, FSH dosage, and also that lower levels of basal testosterone might be related with poor ovarian response^(22)^. Embryo cleavage kinetics were studied by Wissing et al.^(23)^ with a small sample size. Contrary to our results, their study showed slower development to the 8-cell stage from fertilization in patients with hyperandrogenic PCOS^(23)^. An article published in 2014 compared cumulative live birth rates among groups of patients with PCOS, isolated PCO, and controls; their data revealed higher live birth rates in the group with isolated PCO compared with controls. This favorable outcome was not observed in the PCOS group and the authors claimed that this may be due to unfavorable embryo competence otherwise observed in PCOS^(24)^. Again, in their study a general conclusion was drawn from a mixture of patients with different PCOS phenotypes.

The limitations of our study were its retrospective nature and relatively small sample size. The importance of the present study is in the evaluation of assisted reproductive technology (ART) outcomes of different phenotypes of PCOS because there is limited data in the literature that compares subtypes of PCOS in terms of characteristics and outcomes of ART.

## CONCLUSION

To the best of our knowledge, this is a unique study assessing IVF outcomes in different PCOS phenotypes in an acceptable number of participants. PCO morphology provides easier stimulation; hyperandrogenemia provides better results in terms of good quality embryos. Multivariate regression analyses showed that easier stimulation is based on basal FSH level, group, and age. However, the end point was similar regarding biochemical, clinical, and ongoing pregnancy rates.

## Figures and Tables

**Table 1:**
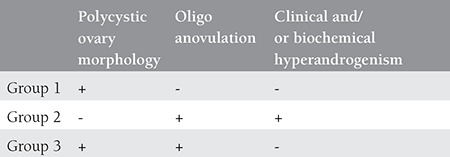
Clinical characteristics of patients according to rotterdam criteria in each group

**Table 2:**
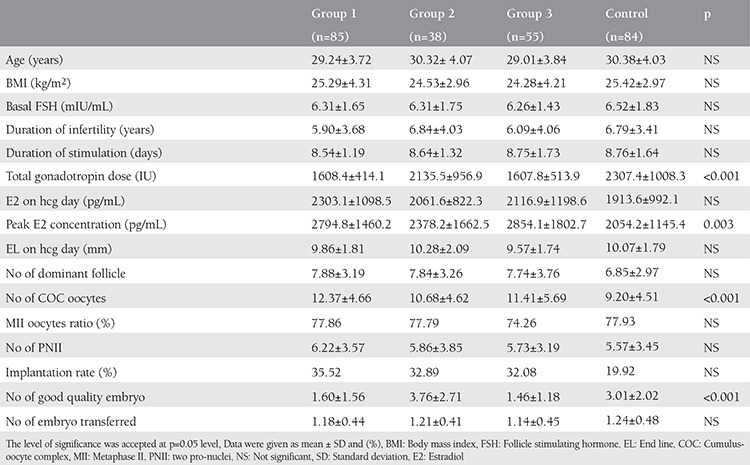
Comparison of baseline clinical characteristics among all groups

**Table 3:**
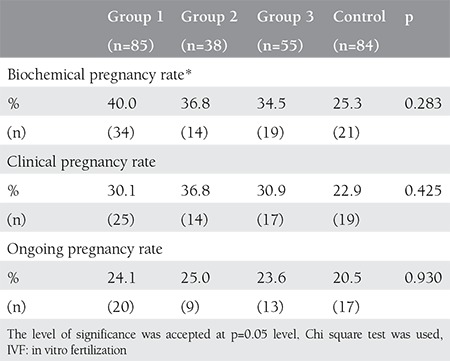
Comparison of outcomes of IVF cycles among all groups
